# Neutropenia Is Not Associated With the Outcomes in Patients With Kawasaki Disease

**DOI:** 10.3389/fped.2021.652346

**Published:** 2021-06-04

**Authors:** Yunjia Tang, Miao Gang, Weiguo Qian, Jin Ma, Qiuqin Xu, Haitao Lv

**Affiliations:** ^1^Department of Cardiology, Children's Hospital of Soochow University, Suzhou, China; ^2^Department of Pharmacy, Children's Hospital of Soochow University, Suzhou, China

**Keywords:** Kawasaki disease, intravenous immunoglobulin resistance, coronary artery lesions, neutrophils, neutropenia

## Abstract

**Objective:** To investigate the outcomes of coronary artery lesions (CALs) and intravenous immunoglobulin (IVIG) resistance in patients with and without neutropenia during the disease course and to explore the relationships between Δ absolute neutrophils count (ΔANC) and the outcomes.

**Methods:** We retrospectively reviewed the medical records of patients hospitalized in Children's Hospital of Soochow University with a main diagnosis of KD during January 2019 and December 2019. 1:4 propensity score matching was carried out to adjust the baseline characteristics. Smoothed plots and threshold effect analyses were performed to reveal the relationships between ΔANC and the outcomes.

**Results:** Of the 438 patients enrolled, 75 (17.1%) were neutropenia cases and 363 (82.9%) were non-neutropenia cases. Patients with neutropenia were younger, had lower levels of initial ANC, white blood cell (WBC) count and C-reactive protein (CRP). Propensity score matching included 75 neutropenia and 247 non-neutropenia patients. No significant difference was found between neutropenia and non-neutropenia groups regarding CALs, coronary artery aneurysms, irregular coronary lumen, IVIG resistance and days of fever duration. There was a non-linear relationship between ΔANC and IVIG resistance. However, threshold effect analysis showed the incidence of IVIG resistance decreased with increasing ΔANC before the turning point (ΔANC = 1.6) (OR = 0.65, 95% CI = 0.50–0.8.4 *P* = 0.001). On the other hand, there was a linear relationship between ΔANC and CALs, even after adjusting the confounders (OR = 1.06, 95% CI = 1.02–1.11, *P* = 0.008).

**Conclusions:** Neutropenia after IVIG was not exactly associated with the outcomes. However, ΔANC was in relation to CALs and IVIG resistance.

## Introduction

Kawasaki disease (KD) also known as mucocutaneous lymph node syndrome, is a commonly seen vasculitis with the predilection for children under 5 years of age (1). It is the main cause of acquired heart diseases in developed countries in recent years due to its cardiac complications. More evidence has shown that KD is caused by the activation of the immune system (2), followed by the activation of several signal pathways (3–5).

Previous studies have revealed that neutrophils play an important role in the pathogenesis of KD in both animal models and autopsy cases (6, 7). Besides, increases of neutrophils in the initial stage are commonly seen in clinical cases and are often considered predictors of adverse outcomes (8, 9). It was reported that delayed apoptosis partly caused the increased number of neutrophils (10) and intravenous immunoglobulin (IVIG), one of the primary treatments, targeted at the apoptosis of neutrophils (11). As a result, some patients developed neutropenia after IVIG infusion. However, little was known about the outcomes of these patients with neutropenia and we speculated if the existence of neutropenia during the disease course played a role in the prognosis regarding coronary artery lesions (CALs), IVIG resistance and the fever durations.

Here, we did a retrospective cohort study to (1) Investigate the outcomes of CALs and IVIG resistance in patients with and without neutropenia during the disease course and (2) Explore the relationships between Δ absolute neutrophils count (ΔANC) and the outcomes.

## Materials and Methods

### Participants

We retrospectively reviewed the medical records of patients hospitalized in Children's Hospital of Soochow University with a main diagnosis of KD during January 2019 and December 2019 including both complete KD and incomplete KD (iKD). The diagnosis of KD was confirmed when a patient was febrile for ≥5 days, together with four of the following five characteristics: (1) Rash, (2) Bilateral conjunctive injection, (3) Cervical lymphadenopathy, (4) Changes of the extremities, (5) Oral mucosal changes. iKD was diagnosed when a patient had two or three compatible clinical characteristics and when other diseases with similar presentations were excluded. All patients were treated with 2 g/kg IVIG in a single dose. In patients who were refractory to initial IVIG, a second dose of IVIG and (or) additional pulse methylprednisolone were administered. The institutional review board of Children's Hospital of Soochow University approved this study (No: 2020CS094).

### Data Collection

Data regarding demographic, clinical, and laboratory characteristics were obtained. These variables included gender, age, days of illness at IVIG initiation, iKD, white blood cell (WBC) count, ANC, serum concentration of albumin, aspartate aminotransferase (AST), alanine aminotransferase (ALT), total bilirubin, and C-reactive protein (CRP). If laboratory tests were performed more than once, we used the highest value for WBC count, AST, ALT, total bilirubin, CRP and the lowest value for serum albumin. Blood tests were routinely performed within the first 24 h on admission and were repeated after defervescence for 72 h, or repeated as appropriate in patients with poor outcomes. Routine two-dimensional echocardiographic evaluation was performed before IVIG initiation and was repeated within 2 weeks of the onset of illness.

### Definitions

Neutropenia was defined as an ANC < 1.5 × 10^9^/L (12). According to severity, we defined neutropenia as severe (ANC < 0.5 × 10^9^/L), moderate (ANC between 0.5 and 1.0 × 10^9^/L) and mild (ANC between 1.0 and 1.5 × 10^9^/L). ΔANC was obtained by initial ANC minus ANC after IVIG. We used the highest value for ANC before IVIG initiation and the lowest value for ANC after IVIG. In patients with IVG resistance, ANC was also collected before additional pulse methylprednisolone. We calculated the z scores of the left main coronary artery, left circumflex artery, left descending artery, and right coronary artery based on the method reported (13). Coronary artery lesions (CALs) were defined as the z score of any coronary artery ≥2.5 and (or) clearly irregularity of the coronary lumen. IVIG resistance was referred to when a patient had a persistent or recrudescent fever >38.0°C lasting for more than 36 h after the initiation of IVIG (1).

### Adjustment for Differences in Baseline Characteristics

Demographic, clinical and laboratory characteristics before IVIG initiation of patients with and without neutropenia were compared. 1:4 propensity score matching using a multivariable logistic regression model was conducted to match the participants between the two groups. Patients in the two groups were matched without replacement on the logit of the estimated propensity score using caliper width 0.20 of the standardized difference (SD) of the logit of the propensity score. We considered an SD < 10% as well-balanced in the baseline characteristics between the two groups.

### Statistical Analysis

Categorical variables were expressed as numbers with percentages and were compared using chi-square test or Fisher's exact-test. Cochran-Mantel-Haenszel tests were used to compare the outcomes of CALs and IVIG resistance, stratified by gender. Pearson correlation between ages and neutrophils count in neutropenia was performed. Continuous variables were shown as median with mean ± standard deviation (SD) or median with quartiles. In the comparisons between the two groups, the Mann–Whitney *U*-test or Student *t*-test was used as appropriate. Paired *t*-test was used to compare ANC before and after initial IVIG treatment. In the comparisons among three groups, the Kruskal Wallis *H*-test was used. Bonferroni *post-hoc* test was used to compare every two groups among three groups.

Sensitivity analyses were also performed. First, logistic regression was carried out including patients before propensity score matching to explore the risk factors of CALs and IVIG resistance. Second, we carried out propensity score matching including hospital identification number as a covariate.

We further applied a two-piecewise linear regression model to examine the threshold effect of the ΔANC on CALs using a smoothing function. The threshold level was determined using trial and error, including selection of turning points along a pre-defined interval and then choosing the turning point that gave the maximum model likelihood. The adjusted confounders of CALs and IVIG resistance were those obtained in the sensitivity analysis of logistic regression. All the statistics were performed by SPSS 22.0 (IBM, Armonk, NY, USA) and Empower (*R*) (www.empowerstats.com, X&Y solutions, Inc. Boston, MA). A two-sided *P* < 0.05 was considered significant.

## Results

**Figure 1:**
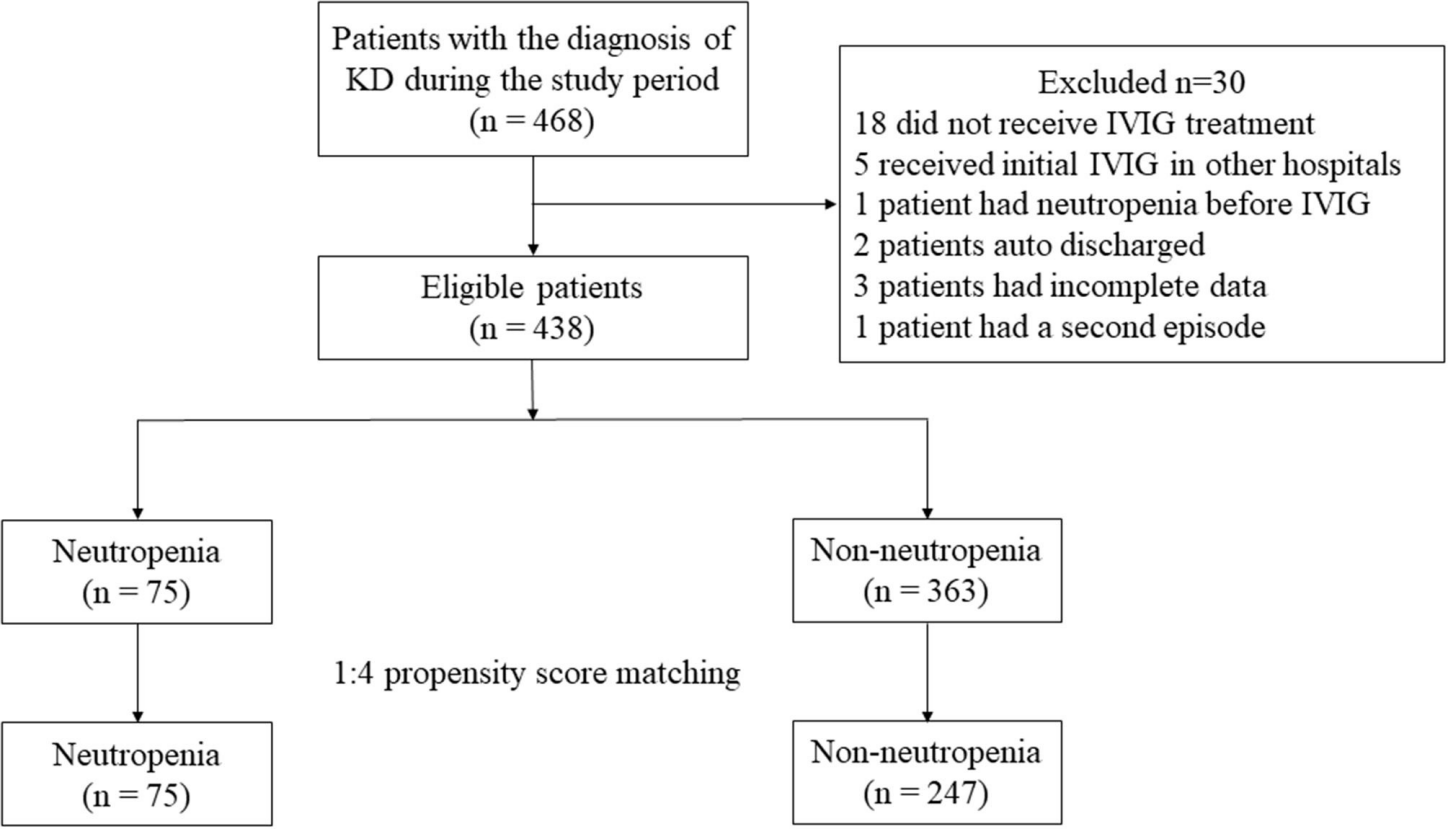
Study flow diagram. KD, Kawasaki disease.

A total of 468 patients were treated with the main diagnosis of KD in our hospital during the study period. Among them, one patient had a second episode in 1 year and we only included the first episode. Eighteen patients did not receive IVIG treatment due to economic concerns or the defervescence before IVIG, and 5 patients received initial IVIG treatment in other hospitals prior to admission. One patient had neutropenia before IVIG, 2 patients auto discharged and 3 patients had incomplete data. As a result, 438 patients including 246 (56.2%) males and 192 (43.8%) females were enrolled in further analysis. Of them, 76 (17.4%) were iKD and 362 (82.6%) were complete KD. Forty (9.1%) cases were termed IVIG resistance. Seventy-five (17.1%) were identified as neutropenia cases and the remaining 363 (82.9%) were identified as non-neutropenia cases (Figure 1). No significant difference was found regarding iKD in neutropenia and non-neutropenia cases (21.3 vs. 16.5%, *P* = 0.317).

All patients with IVIG resistance received additional treatments including pulse methylprednisolone in two, a second dose of IVIG together with pulse methylprednisolone in 35, a third dose of IVIG together with pulse methylprednisolone in three. The highest ANC before initial IVIG was obtained at a median of the 5th day of disease onset (mean ± SD: 4.5 ± 1.8 days) while the lowest ANC after defervescence was obtained at a median of the 15th day of disease onset (mean ± SD: 17.4 ± 6.3 days) in patients with IVIG resistance. We also compared ANC before initial IVIG with ANC before additional treatments in these patients. Blood routine tests before additional treatments were carried out in 25 (62.5%) patients. ANC before initial IVIG and before additional treatments were [10.6 (8.3, 14.6) vs. 7.5 (4.8, 11.4) × 10^9^/L, respectively, *P* = 0.264]. On the other hand, ANC before and after initial IVIG in patients without IVIG resistance were [9.6 (6.6, 13.5) vs. 2.7 (1.8, 4.1) × 10^9^/L, *P* < 0.001].

**Figure 2:**
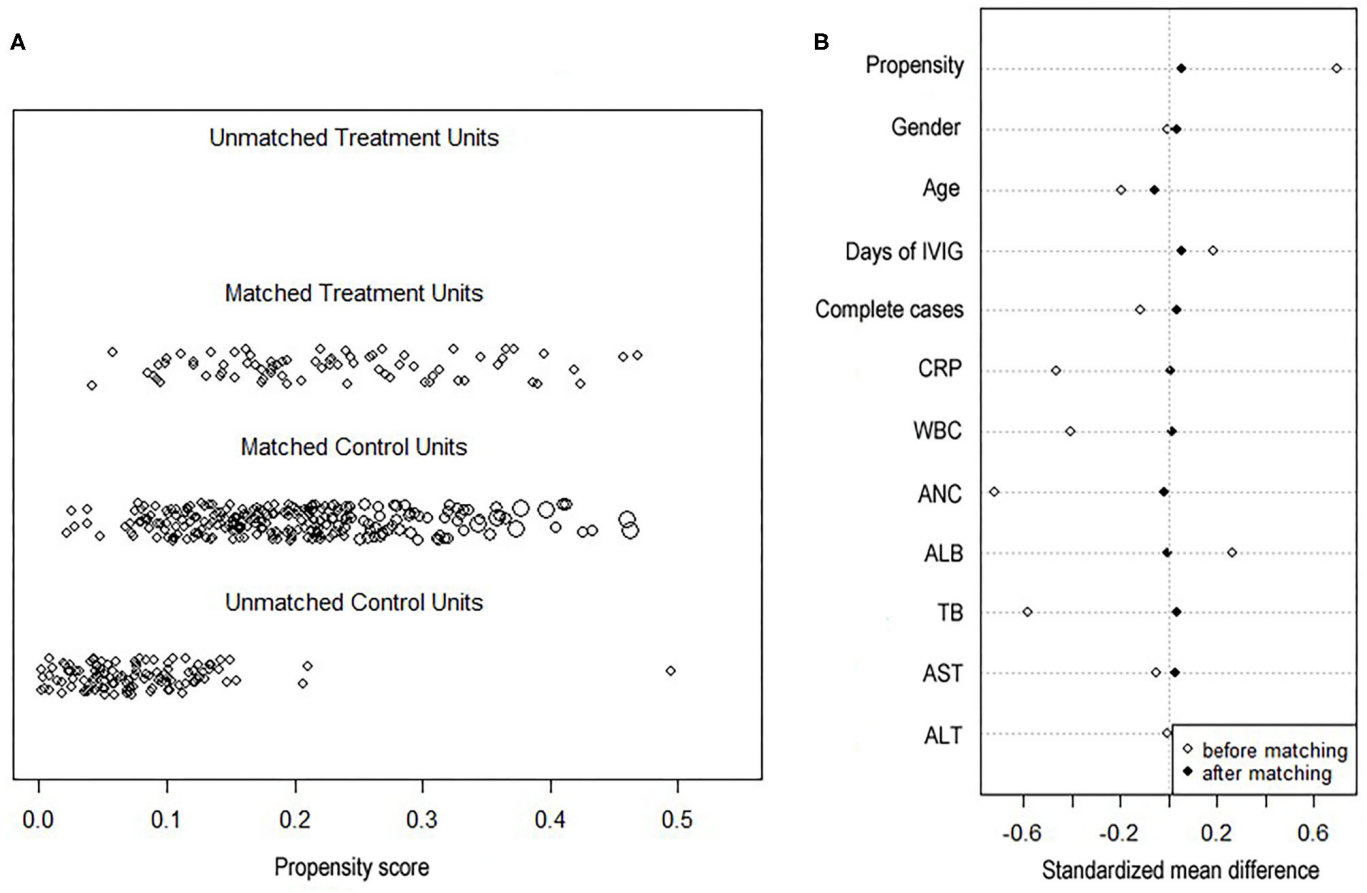
Propensity score plots. **(A)** Jitter plot of propensity score distribution of individual cases. **(B)** Love plot of standardized mean differences comparing baseline characteristics of neutropenia and non-neutropenia before and 1:4 after propensity score matching. Y axis was the baseline characteristics. X axis of the scatterplot represented whether the status was before-matching or after matching. CRP, C-reactive protein; WBC, white blood cell; ANC, absolute neutrophils count; ALB, serum albumin; AST, aspartate aminotransferase; ALT, alanine aminotransferase.

A 4:1 propensity score matching pair was created including 75 neutropenia and 247 non-neutropenia patients. Figure 2A shows Jitter plot of propensity score distribution of individual cases. The absolute SDs of all the variables were lower than 0.1, indicating a well-balanced matching between the two groups (Figure 2B). The baseline characteristics before and after the propensity score matching are shown in Table 1. Patients with neutropenia were younger, had lower levels of initial neutrophils count, WBC count and CRP (*P* < 0.05). Days of illness at IVIG initiation were longer, serum albumin was higher and total bilirubin was lower in patients with neutropenia, although these variables were not statistically significant.

**Table 1:** Characteristics of patients pre and post-propensity score matching.

**Variables**	**Pre-propensity score–matched patients**			**Post-propensity score–matched patients**		
	**Neutropenia**	**Non-neutropenia**	***P***	**Neutropenia**	**Non-neutropenia**	**SD**
Number, *n*	75	363	-	75	247	-
Age, months, median (quartile)	20 (12, 35)	27 (15, 46)	0.017	20 (12, 35)	25 (14, 41)	−0.057
Gender, male (%)	42 (56.0)	204 (56.2)	0.975	42 (56.0)	137 (55.5)	0.031
Days of illness at IVIG initiation, median (mean ± SD)	7.0 (7.7 ± 2.3)	7.0 (7.2 ± 1.9)	0.053	7.0 (7.7 ± 2.3)	7.0 (7.3 ± 1.9)	0.055
Incomplete KD, *n* (%)	16 (21.3)	60 (16.5)	0.317	16 (21.3)	51 (20.6)	0.032
**Laboratory data before IVIG initiation, median (quartiles)**						
CRP, mg/dl	57.1 (30.0, 89.5)	71.4 (42.2, 112.0)	0.003	57.1 (30.0, 89.5)	61.0 (34.6, 89.0)	0.011
ANC, × 10^9^/L	7.8 (4.9, 10.5)	10.3 (6.9, 13.9)	<0.001	7.8 (4.9, 10.5)	8.5 (6.3, 11.4)	−0.019
WBC, × 10^9^/L	12.8 (8.6, 16.2)	15.0 (11.5, 19.8)	<0.001	12.8 (8.6, 16.2)	13.3 (10.6, 17.4)	0.017
Serum albumin, g/L	39.7 (36.9, 42.2)	39.4 (36.8, 41.1)	0.086	39.7 (36.9, 42.2)	39.7 (37.8, 41.4)	−0.006
Total bilirubin, μmol/L	5.1 (3.4, 8.0)	5.9 (4.1, 9.8)	0.053	5.1 (3.4, 8.0)	5.1 (3.7, 7.7)	0.036
AST, U/L	31.5 (25.3, 54.4)	24.1 (12.9, 73.6)	0.931	31.5 (25.3, 54.4)	31.9 (24.5, 46.3)	0.025
ALT, U/L	33.1 (25.0, 49.5)	27.8 (13.3, 84.7)	0.609	33.1 (25.0, 49.5)	23.0 (12.5, 54.7)	0.057

*SD, Standardized difference; CRP, C-reactive protein; ANC, absolute neutrophils count; WBC, white blood cell; AST, aspartate aminotransferase; ALT, alanine aminotransferase*.

After propensity score matching, we compared the outcomes of CALs, IVIG resistance, and days of total fever duration. The results are shown in Table 2. No significant difference was found between neutropenia and non-neutropenia groups regarding CALs, coronary artery aneurysms, irregular coronary lumen, IVIG resistance, and days of fever duration. Two giant coronary artery aneurysms were documented in these patients.

**Table 2:** Comparisons of outcomes in neutropenic and non-neutropenic patients after propensity score matching.

	**Neutropenia**	**Non-neutropenia**	***P***
Number, *n*	75	247	-
CALs, *n* (%)	24 (32.0)	56 (22.7)	0.102
Coronary artery aneurysms	16 (21.3)	39 (15.8)	0.264
Irregular coronary lumen	20 (26.6)	46 (18.6)	0.131
Giant coronary artery aneurysms	1 (1.3)	1 (0.4)	0.412
IVIG resistance, *n* (%)	2 (2.6)	21 (8.5)	0.086
Fever duration, days	7.0 (7.8 ± 2.4)	7.0 (7.5 ± 2.0)	0.271

*CALs, coronary artery lesions*.

We further grouped neutropenia into three categories of severe, moderate, and mild. There were no differences in all of the baseline characteristics but gender. Neutrophil counts were not correlated to ages (*P* = 0.252). A significant difference was found in irregular coronary lumen (*P* < 0.05, Table 3). However, the significances became statistically insignificant after Bonferroni *post-hoc* test. Cochran-Mantel-Haenszel test showed significant difference in males regarding irregular coronary lumen (*P* = 0.021), and the difference between mild and moderate neutropenia remained significant even after Bonferroni *post-hoc* test (*P* = 0.016, which was lower than 0.017), that was patients with moderate neutropenia had higher incidences of irregular coronary lumen than patients with mild neutropenia in males.

**Table 3:** Subgroup analysis of neutropenic patients.

	**Severe**	**Moderate**	**Mild**	***P***
Number, *n*	7	21	47	-
**Baseline characteristics**				
Age, months, median (quartile)	13.0 (5.0, 18.0)	29.0 (11.5, 35.5)	19.0 (13.0, 36.0)	0.059
Gender, male (%)	7 (100)	10 (47.6)	25 (53.2)	0.036
Days of illness at IVIG initiation, median (mean ± SD)	8.0 (8.3 ± 2.4)	7.0 (7.9 ± 3.2)	7.0 (7.4 ± 1.7)	0.764
Incomplete KD, *n* (%)	3 (42.9)	4 (19.0)	9 (19.1)	0.390
**Laboratory data before IVIG initiation, median (quartiles)**				
CRP, mg/dl	38.7 (13.0, 90.6)	57.3 (27.2, 94.0)	57.8 (30.5, 91.4)	0.735
ANC, × 10^9^/L	7.9 (3.0, 11.6)	6.4 (4.5, 9.7)	8.3 (5.4, 10.5)	0.305
WBC, × 10^9^/L	14.4 (6.3, 17.5)	11.9 (8.0, 15.9)	13.1 (9.3, 16.2)	0.615
Serum albumin, g/L	40.9 (36.8, 42.5)	39.1 (36.6, 42.0)	39.6 (37.2, 42.1)	0.660
Total bilirubin, μmol/L	3.0 (2.0, 12.7)	5.3 (3.8, 9.6)	5.0 (3.4, 7.6)	0.374
AST, U/L	45.1 (31.7, 91.0)	31.7 (22.3, 70.4)	28.8 (24.6, 44.3)	0.191
ALT, U/L	25.6 (17.5, 72.3)	32.7 (10.2, 110.4)	23.1 (12.8, 74.0)	0.838
**Outcomes**				
CALs, *n* (%)	3 (42.9)	9 (42.9)	12 (25.5)	0.263
Coronary artery aneurysms	3 (42.9)	5 (23.8)	8 (17.0)	0.239
Irregular coronary lumen	3 (42.9)	9 (42.9)	8 (17.0)	0.039
IVIG resistance, *n* (%)	0 (0)	1 (4.8)	1 (2.1)	0.610
Fever durations, days	8.0 (8.3 ± 2.5)	7.0 (8.0 ± 3.5)	7.0 (7.7 ± 1.8)	0.639

*CALs, coronary artery lesions; IVIG, intravenous immunoglobulin; CRP, C-reactive protein; ANC, absolute neutrophils count; WBC, white blood cell; AST, aspartate aminotransferase; ALT, alanine aminotransferase*.

Sensitivity analyses were carried out. First, a forward stepwise multivariate logistic regression analysis was conducted. The results are shown in Tables 4, 5. Neutropenia was not selected in the regression model to indicate CALs or IVIG resistance (*P* = 0.234 and 0.069, respectively). Second, we enrolled hospital identification number as a covariate during propensity score matching, we found the same results regarding CALs (OR:1.600, 95% CI: 0.905–2.828, *P* = 0.106), IVIG resistance (OR:0.147, 95% CI: 0.091–1.115, *P* = 0.064) and total fever duration [(7.8 ± 2.4) vs. (7.6 ± 2.0) days, *P* = 0.363].

**Table 4:** Multivariate indicators of CALs in Kawasaki disease.

**Variables**	**Logistic coefficient**	**OR (95% CI)**	**Wald**	***P***
Gender	−0.039	0.962 (0.948–0.976)	26.876	<0.001
Days of illness at IVIG initiation	0.199	1.220 (1.078–1.380)	9.874	0.002
Serum albumin	−0.091	0.913 (0.862–0.967)	9.577	0.002
Total bilirubin	0.027	1.027 (1.009–1.046)	8.722	0.003

*CALs, coronary artery lesions*.

**Table 5:** Multivariate indicators of IVIG resistance in Kawasaki disease.

**Variables**	**Logistic coefficient**	**OR (95% CI)**	**Wald**	***P***
Gender	−0.028	0.972 (0.950–0.994)	6.016	0.014
Days of illness at IVIG initiation	−0.436	0.647 (0.491–0.853)	9.534	0.002
Serum albumin	−0.167	0.846 (0.769–0.931)	11.799	0.001
Total bilirubin	0.033	1.033 (1.000–1.003)	9.580	0.002

*IVIG, intravenous immunoglobulin*.

**Figure 3:**
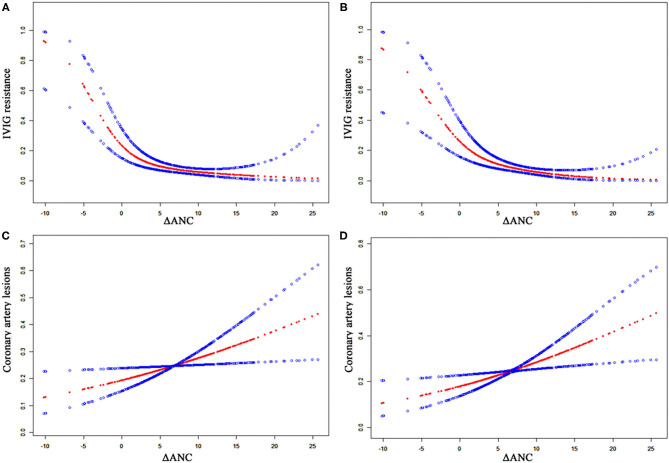
Relationship between ΔANC and the outcomes of CALs and IVIG resistance. The blue dots indicate 95% confidence interval. **(A)** ΔANC and IVIG resistance without adjustment. **(B)** ΔANC and IVIG resistance, adjusted for gender, days of illness at IVIG initiation, serum albumin, and total bilirubin. **(C)** ΔANC and CALs without adjustment. **(D)** ΔANC and CALs, adjusted for gender, days of illness at IVIG initiation, serum albumin, total bilirubin, and IVIG resistance. ANC, absolute neutrophils count; CALs, coronary artery lesions; IVIG, intravenous immunoglobulin.

Patients with CALs had relatively higher initial ANC than those without CALs [(11.2 ± 5.4) vs. (10.4 ± 5.4) × 10^9^/L]. However, the difference was not statistically significant (*P* = 0.111). Given that initial ANC was significantly higher in non-neutropenia patients, we further analyzed the relationship between ΔANC and the outcomes before propensity score matching (Table 6 and Figure 3). There was a non-linear relationship between ΔANC and IVIG resistance (Figure 3A). After adjusting the confounders (albumin, gender, days of illness at IVIG initiation, and total bilirubin), the result remained the same (Figure 3B). Threshold effect analysis showed the incidence of IVIG resistance decreased with ΔANC before the turning point (ΔANC = 1.6) (OR = 0.65, 95% CI = 0.50–0.84, *P* = 0.001). On the other hand, smoothed plots suggested a linear relationship between ΔANC and CALs (OR = 1.05, 95% CI = 1.01–1.09, *P* = 0.022, Figure 3C), even after adjusting the confounders (albumin, gender, days of illness at IVIG initiation, total bilirubin, and IVIG resistance) (OR = 1.06, 95% CI = 1.02–1.11, *P* = 0.008, Figure 3D).

**Table 6:** Threshold effect analysis of ΔANC on CAL and IVIG resistance using twopiece-wise linear regression.

	**Crude β/OR (95% CI), *P***	**Adjusted β/OR (95% CI), *P***
**CALs**		
ΔANC	1.05 (1.01, 1.09), 0.022	1.06 (1.02, 1.11), 0.008
**IVIG resistance**		
ΔANC < 1.6	-	0.65 (0.50–0.84), 0.001
ΔANC > 1.6	-	0.90 (0.81–1.01), 0.064

*CALs were adjusted for gender, days of illness at IVIG initiation, serum albumin, and total bilirubin. IVIG resistance was adjusted for gender, days of illness at IVIG initiation, serum albumin, and total bilirubin. CALs, coronary artery lesions; ANC, absolute neutrophils count; IVIG, intravenous immunoglobulin*.

## Discussion

Our study showed that neutropenia after IVIG was not exactly associated with the outcomes. However, ΔANC was in relation to the occurrences of CALs and IVIG resistance. Our findings indicated that more attention should be paid to patients with lower ΔANC to be aware of IVIG resistance.

Neutrophils are the most abundant leukocytes in the circulation and play a fundamental role in the innate immune response. Activation of neutrophils is commonly seen in bacterial infections and inflammatory autoimmune diseases. KD as one of the most common autoimmune vasculitis in children also showed abnormally increased neutrophils in varying degrees in the acute stage in our present study. It was widely considered that increased neutrophils played a pivotal role in KD, especially in IVIG resistance (8, 9) and CALs formations (14, 15), although the precise mechanisms were unknown.

However, a certain proportion of patients (17.1%) developed neutropenia after IVIG treatment, which was in line with a previous study (16). They found 17–40% of patients developed neutropenia within the first week after IVIG treatment. It was also pointed out that early neutropenia within the 10th day of disease onset played an important role in the prevention of CALs (16). In contrast, we found no significant differences in CALs in patients with and without neutropenia with baseline equilibrium after 1:4 propensity score matching, which was in line with Wang's study, although they set the time of CALs at a 3-month follow-up (17). The divergence was caused by different study designs, as we did not specifically focus on the time of neutropenia onset. On the other hand, no significant differences were found regarding IVIG resistance and the length of fever durations, indicating the existence of neutropenia might not be an indication of worse or better outcomes.

Interestingly, all severe neutropenia cases were males and males with moderate neutropenia were more likely to have irregular coronary lumen than those with mild neutropenia in the subgroup analysis. When the basal neutrophil counts were reported to be almost the same in males and females in healthy children (18), it was hard to tell whether males were prone to developing severe neutropenia as they were more vulnerable to KD, or the phenomenon was caused by coincidence. In this case, further prospective studies are necessary to confirm our results.

We found ΔANC was related to IVIG resistance in a linear relationship when ΔANC was lower than 1.6, indicating that the lower ΔANC was, the higher incidence of IVIG resistance there was. Moreover, ANC did not decrease significantly after initial IVIG in patients with IVIG resistance. IVIG appeared to have a generalized anti-inflammatory effect. Although its specific mechanisms in KD remained obscure, it was considered that IVIG played a role by interacting with components of the immune system (19), such as macrophage/monocytes, dendritic cells, antibodies, T cells, NK cells and neutrophils. Previously, IVIG was found to mediate neutrophils death *in vitro* (11, 20). Thus, we hypothesized that IVIG resistance was partly related to an insufficient death of neutrophils and a lower ΔANC was indicative of IVIG resistance.

Although the risk factors of CALs were widely explored all over the world, which were mainly focused on the demographic and initial laboratory characteristics (21–24), ΔANC was seldom studied (17). Interestingly, we found higher ΔANC was indicative of CALs. While lower ΔANC was associated with IVIG resistance, which was widely considered as a risk factor of CALs, a relatively higher initial ANC in patients with CALs might partly explain it. Otherwise, some other confounders might also play roles. Our results were not totally in line with a previous study, in which the authors showed patients with ΔANC larger than 6 had greater CALs rates (17). We attributed the divergence to the following reasons. First, we had different definitions of CALs. Second, the treatment regimens in their study were not the same as ours. In our study, a single dose of 2 g/kg IVIG was administered as recommended by the latest guideline (1), and corticosteroids were routinely used in patients with IVIG resistance. Most importantly, we further excluded several confounders including IVIG resistance to explore the relationship between ΔANC and CALs.

The present study had some limitations. First, the echocardiographic results during the subacute and the convalescent stage were not collected and analyzed, because most of the CALs occurred during the early stage of the disease (25). However, it should be further investigated to give an overview. Second, a small number of neutropenic patients were enrolled in the subgroup analysis, which might lead to a bias. Third, the baseline numbers of neutrophils were not in line with each other in different age groups (18), and younger children tended to have lymphocytes dominance. We did not classify neutropenia according to the ages in the present study, because there were no such definitions of age-specific neutropenia. Fourth, it was reported that the time of neutropenia onset also counted in the CALs formations (16). However, we were unable to explore it because the blood routine test was not daily performed. Last, corticosteroids were routinely used in patients with IVIG resistance, which would possibly lead to an increase in neutrophils. When we analyzed ANC before initial IVIG and before additional pulse methylprednisolone, ANC before methylprednisolone was only available in 62.5% of the patients. Overall, the results of the study were robust for a relatively large number of participants with baseline equilibrium and were strengthened by sensitivity analyses.

## Data Availability Statement

The original contributions presented in the study are included in the article/supplementary material, further inquiries can be directed to the corresponding authors.

## Author Contributions

YT wrote the manuscript. MG collected the data. WQ and JM analyzed and interpreted the data. QX and HL designed the study and reviewed the manuscript. All authors read and approved the final manuscript.

## Conflict of Interest

The authors declare that the research was conducted in the absence of any commercial or financial relationships that could be construed as a potential conflict of interest.

## Abbreviations

KD: Kawasaki disease
CALs: coronary artery lesions
IVIG: intravenous immunoglobulin
ANC: absolute neutrophils count
iKD: incomplete KD
CRP: C-reactive protein
WBC: white blood cells
ALT: alanine aminotransferase
AST: aspartate aminotransferase
SD: standardized difference
OR: odd ratio
CI: confidence interval.
